# Supplementary material for: Global H3.3 dynamic deposition defines its bimodal role in cell fate transition
Source: Nat Commun. 2018 Apr 18;9:1537. doi: 10.1038/s41467-018-03904-7 (PMC5906632; doi:10.1038/s41467-018-03904-7)
Supplement: Supplementary file 1 — Supplementary Information [file 41467_2018_3904_MOESM1_ESM.pdf]

Supplementary Information

Global H3.3 dynamic deposition defines its bimodal role in cell fate transition

Fang et al.

Supplementary Figure 1

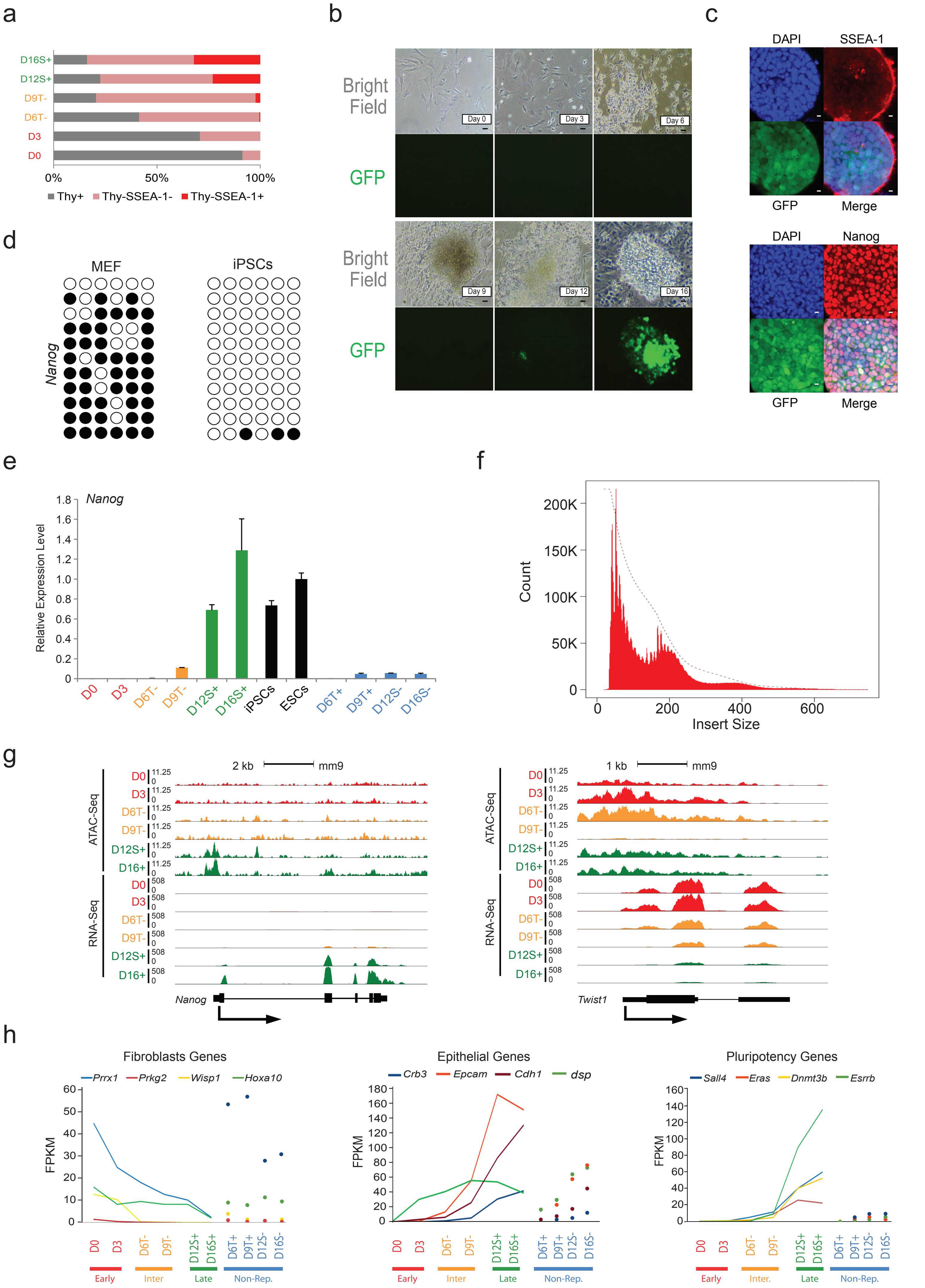

Supplementary Figure 1: (Related to Figure 1). Reprogrammed iPSCs demonstrate acquisition of pluripotency.

(a) Percentage stacked columns demonstrating the abundance levels (x-axis) of Thy+, Thy-SSEA1- and Thy-SSEA-1+ cells at the indicated time-points (Y-axis).

(b) Representative microscopic images of cells at different stages of cellular reprogramming under bright field and fluorescence (green – GFP). Scale bars equal 100 µm.

(c) Representative microscopic images of iPSCs stained with DAPI (blue), SSEA-1 (top - red) and Nanog (bottom – red). Green GFP indicates the expression of endogenous Oct4. Scale bars equal 100 µm.

(d) Methylation profile of *Nanog* promoter in MEFs (left) and iPSCs (right). Black denotes methylation whereas white represents no methylation.

(e) Bar chart revealing the relative expression level (y-axis) of *Nanog* in cells undergoing reprogramming at the time-points indicated (x-axis). The expression values are calculated relative to the expression level of *Nanog* in mESCs. Values are mean ± s.e.m from independent replicate experiments (n = 3). Error bars represent standard deviation.

(f) Nucleosomal distribution patterns observed in the ATAC-Seq libraries.

(g) UCSC screenshot demonstrating the dynamic accessibility and expression of *Nanog* (left) and *Twist1* (right) during cellular reprogramming.

(h) Line plots demonstrating the dynamic expression of the indicated fibroblast (left), epithelial (middle) and pluripotency (right) genes during cellular reprogramming. Y-axis represents the FPKM values of these genes at the indicated time-points in (x-axis).

Supplementary Figure 2

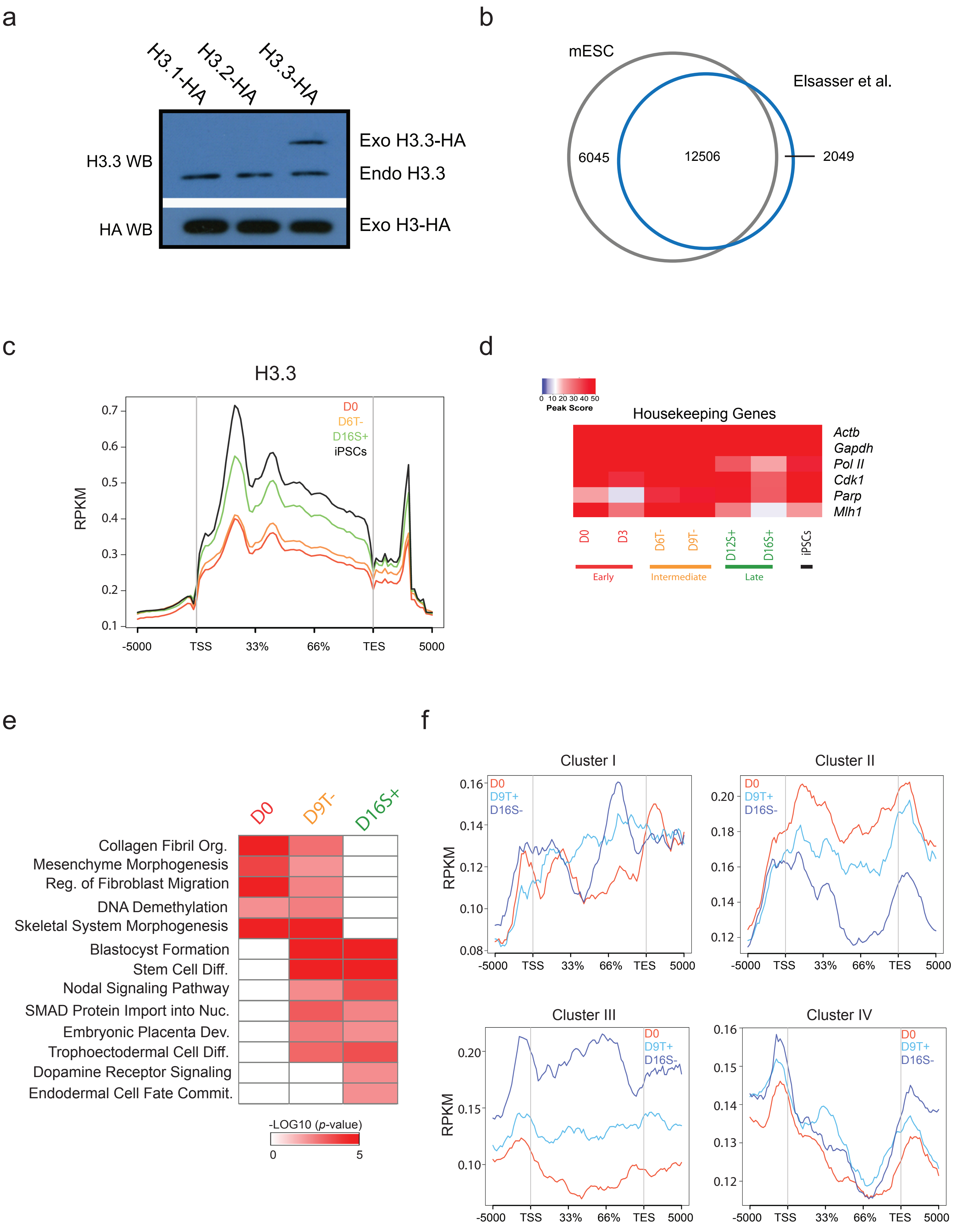

Supplementary Figure 2: (Related to Figure 2). Profiles of H3.3 genomic deposition during reprogramming.

(a) H3.3 western blots for cells with overexpressed H3.1, H3.2 or H3.3.

(b) Venn diagram demonstrating the number of uniquely and commonly bound genes between published H3.3 ChIP-Seq library and mESC H3.3 library prepared in this study.

(c) Average enrichment profile of H3.3 reads (around genebodies) for the indicated time-points. The Y-axis represents average normalized number of fragments mapping to the corresponding regions indicated in the x-axis.

(d) Heatmap revealing the dynamic enrichment of H3.3 over the indicated housekeeping genes. The values represent peak scores calculated by findPeaks script and the colour ranges from dark blue (low enrichment) to dark red (high enrichment).

(e) Differential GO analysis revealing specifically enriched biological processes by genes bound by H3.3 at D0, D9T- and D16S+. The colour ranges from white (no enrichment) to dark red (high enrichment).

(f) Average enrichment profile of H3.3 reads, belonging to the libraries at the indicated time-points, around the genebodies of Cluster I (top left), Cluster II (top right), Cluster III (bottom left) and Cluster IV (bottom right) genes. The Y-axis represents average normalized number of fragments mapping to the corresponding regions indicated in the x-axis.

Supplementary Figure 3

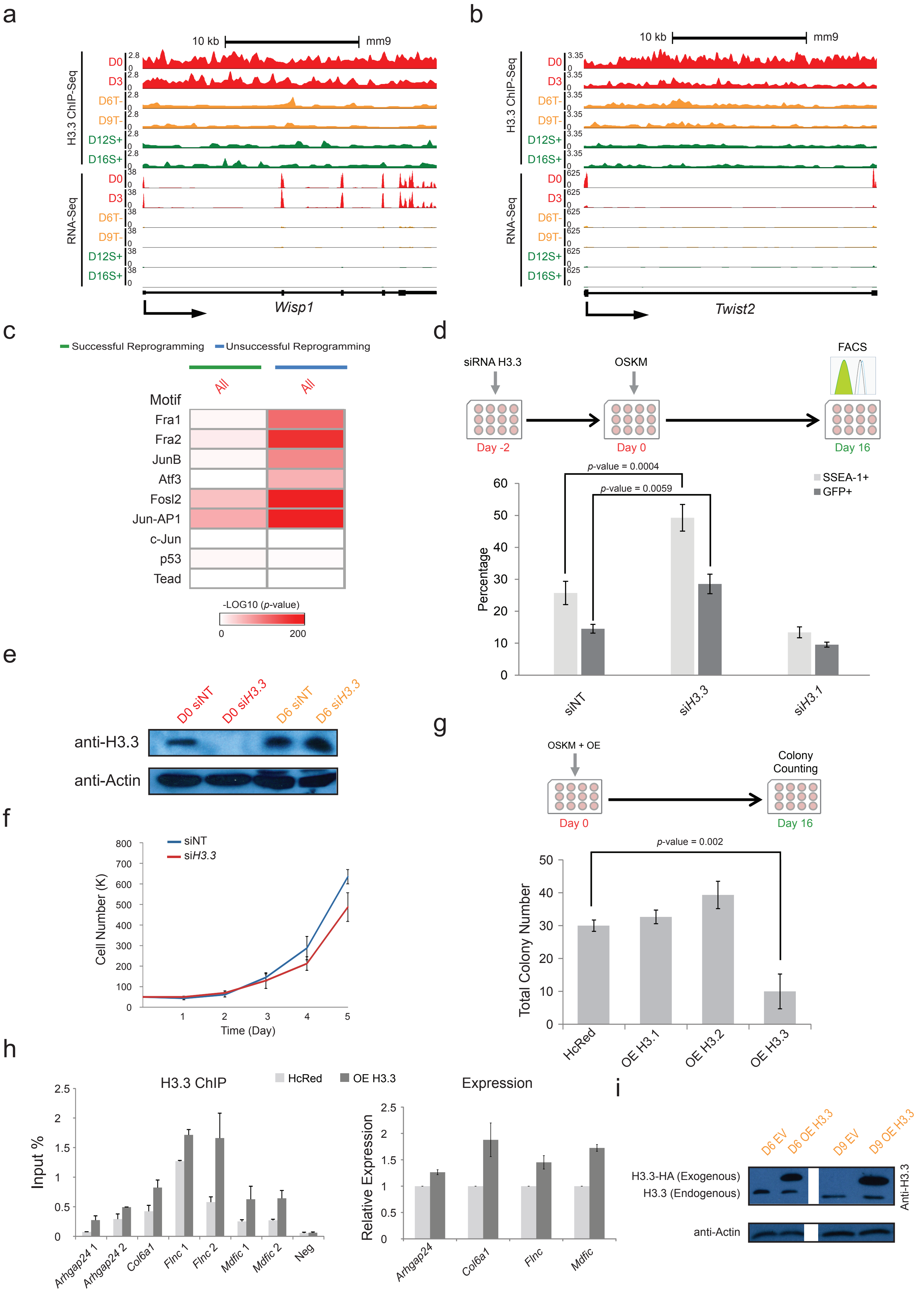

Supplementary Figure 3: (Related to Figure 3). Removal of H3.3 from fibroblast lineage genes is required for successful reprogramming.

(a) UCSC screenshots demonstrating the enrichment level of H3.3 on *Wisp1* (fibroblast gene). The dynamic expression level (RNA-Seq) can also be seen in the screenshot.

(b) UCSC screenshots indicating the dynamic enrichment level of H3.3 on *Twist2* (mesenchymal gene). The expression level (RNA-Seq) can also be seen in the screenshot.

(c) Heatmap revealing the enrichment of the motifs of the indicated transcription factors in the intergenic regions that retained H3.3 binding in the successful (green) and unsuccessful (blue) routes. Enrichment ranges from not enriched (white) to highly enriched (dark red).

(d) Schematics of the siH3.3 knockdown experiment (top). The bar chart below represents the percentage of SSEA+ cells (light grey) and GFP+ cells (dark grey). Non-targeting siRNA constructs (siNT) were used as controls. Values are mean ± s.e.m from independent replicate experiments.

(e) Western blots of H3.3 in day 0 and day 6 cells in which *H3.3* expression was depleted two days prior to reprogramming. Non-targeting siRNA constructs (siNT) were used as controls.

(f) Line plot demonstrating the proliferation of fibroblast cells in which H3.3 was knocked-down. Non-targeting siRNA constructs (siNT) were used as controls. Y-axis denotes the cell number whereas the x-axis represents the time-point. Values are mean ± s.e.m from independent replicate experiments.

(g) Schematics of the H3 histone variants overexpression experiment (top). The bar chart below represents the number of induced pluripotent cells colonies (y-axis) observed in wells with the overexpression of the indicated constructs (x-axis). Cells overexpressed with empty vectors were used as controls (HcRed). Values are mean ± s.e.m from independent replicate experiments.

(h) Bar chart demonstrating the level of H3.3 enrichment on the genes indicated in the x-axis upon the overexpression of H3.3 (H3.3 OE). Empty expression vectors (HcRed) were used as controls. Y-axis represents the enrichment level in the ChIP sample over input (left). Bar chart demonstrating the expression levels of the same genes upon the overexpression of H3.3 (H3.3 OE). Empty expression vectors (HcRed) were used as controls. Y-axis represents the relative expression levels (right). Values are mean ± s.e.m from independent replicate experiments.

(i) Western blots of H3.3 in day 6 and day 9 cells in which the overexpression of H3.3 was induced at the beginning of the cellular reprogramming. Empty vectors (EV) were used as control.

Supplementary Figure 4

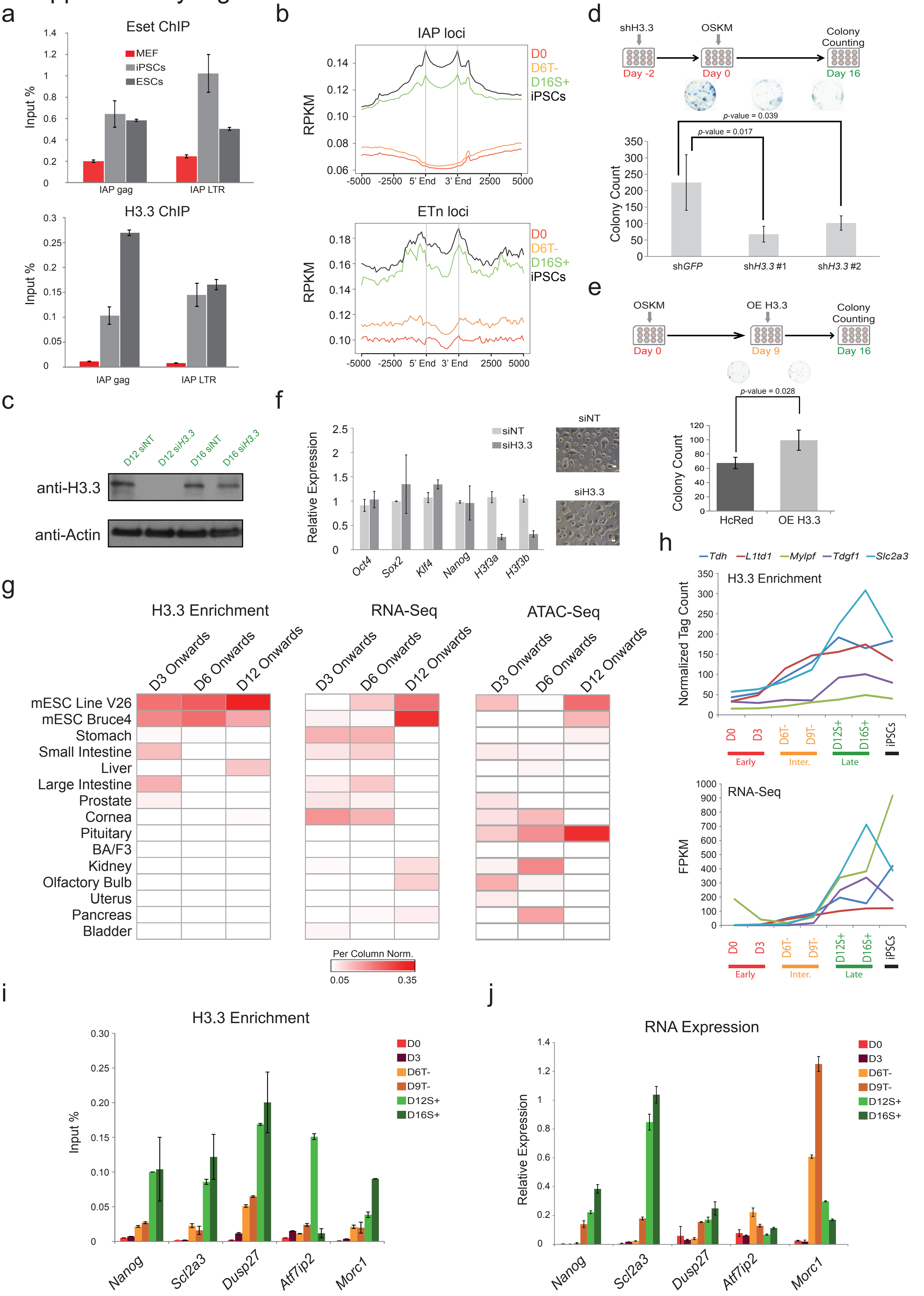

Supplementary Figure 4: (Related to Figure 4). The binding profile of H3.3 in cells at intermediate and later stages of reprogramming.

(a) Bar charts demonstrating the binding level of Eset (above) and H3.3 (below) on IAP gag and IAP LTR in MEFs, iPSCs and ESCs. Y-axis represents the percentage of enrichment over input. Values are mean ± s.e.m from independent replicate experiments (n = 3). Error bars represent standard deviation.

(b) Average enrichment profile of H3.3 reads of the indicated time-points, around IAP genomic loci (above) and ETn genomic loci (below). The Y-axis represents average normalized number of fragments mapping to the corresponding regions indicated in the x-axis.

(c) Western blots of H3.3 in day 12 and day 16 reprogramming cells in which *H3.3* expression was depleted at day 9 of the reprogramming. Non-targeting siRNA constructs (siNT) were used as controls.

(d) Schematics of the constitutive knockdown experiment (top). The bar chart below represents the number of colonies (y-axis) observed in wells. The images above the bar chart are representative images of the wells. Values are mean ± s.e.m from independent replicate experiments (n = 3). Two-tailed t-test was used for statistical analysis. Error bars represent standard deviation.

(e) Schematics of the later time-point overexpression experiment (top). The bar chart below represents the number of colonies (y-axis) observed in wells in which overexpression of H3.3 had been induced. The images above the bar chart are representative images of the wells in which the counting took place. Values are mean ± s.e.m from independent replicate experiments (n = 3). Two-tailed t-test was used for statistical analysis. Error bars represent standard deviation.

(f) Bar chart revealing the relative expression levels (y-axis) of the indicated genes (x-axis) in mESCs in which H3.3 was depleted. Values are mean ± s.e.m from independent replicate experiments (n = 3). Error bars represent standard deviation. (left). Representative images of E14 mESCs colonies in which H3.3 levels were depleted (siH3.3) and WT E14 cells (siNT) (right). Scale bars equal 100 µm.

(g) Heatmaps revealing cell types enrichment from D3, D6 or D12 onwards in H3.3 ChIP-Seq (left), RNA-Seq (middle) and ATAC-Seq (right). The values are per-column normalized hypergeometric enrichment scores for each cell type. The scale ranges from white (no enrichment) to dark red (highly enriched).

(h) Line plots demonstrating the dynamic enrichment of H3.3 (above) and expression levels (below) on the indicated D3 onwards genes during cellular reprogramming. Y-axis represents the normalized number of reads mapping to the genebodies of these genes at the indicated time-point in the x-axis.

(i) Bar chart demonstrating the binding level of H3.3 on the indicated genes (x-axis) in cells undergoing reprogramming at the indicated time-points. Y-axis represents the percentage of enrichment over input. Values are mean ± s.e.m from independent replicate experiments (n = 3). Error bars represent standard deviation.

(j) Bar chart demonstrating the relative expression levels of the indicated genes in the x-axis in cells undergoing reprogramming at the indicated time-points. Y-axis represents the relative expression levels. Values are mean ± s.e.m from independent replicate experiments (n = 3). Error bars represent standard deviation.

Supplementary Figure 5

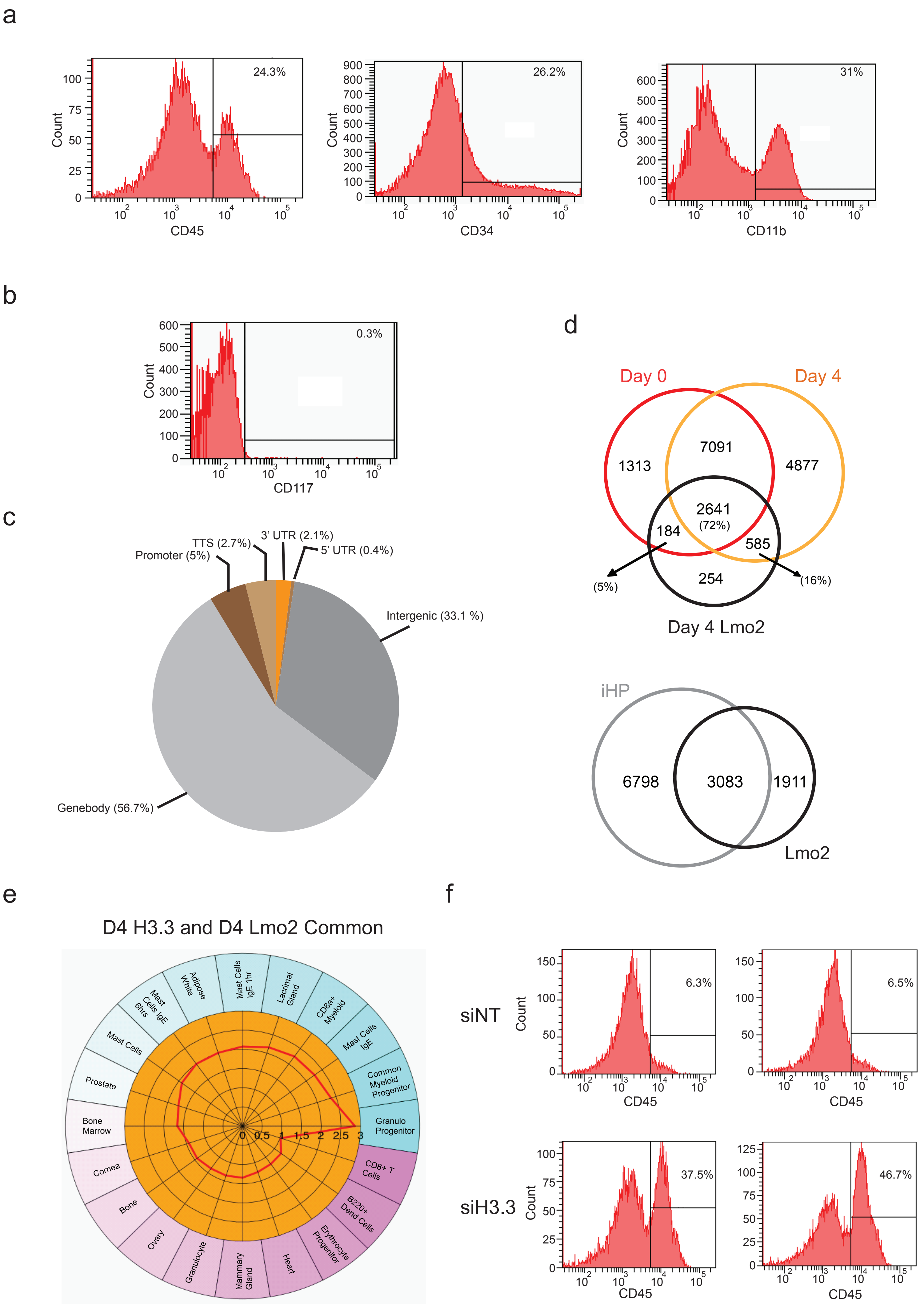

Supplementary Figure 5: (Related to Figure 5). Role of H3.3 in fibroblast transdifferentiation to iHPs.

(a) FACS analysis of suspension cells on day 25 of fibroblast transdifferentiation using CD45 (left), CD34 (middle) and CD11b (right) as markers.

(b) FACS analysis of suspension cells on day 25 transdifferentiation using CD117 (right) as marker.

(c) Average distribution of H3.3 peaks on the indicated genomic regions during transdifferentiation.

(d) Venn diagrams revealing the number of uniquely and commonly bound genes among day 0 H3.3, day 4 H3.3 with day 4 Lmo2 (top) and iHP H3.3 with Lmo2 (bottom).

(e) Cell type enrichment analysis for genes which are commonly bound between Day 4 H3.3 and Day 4 Lmo2. The enrichment score given to each cell type, shown in the circle, represents the hypergeometric score of each cells (-Log10(*p*-value)).

(f) FACS analysis of all H3.3-depleted cells in wells at day 25 of the transdifferentiation process using CD45 as marker. Non-targeting siRNA constructs (siNT) were used as controls. The percentage of CD45+ cells are shown in the histograms.

Supplementary Figure 6

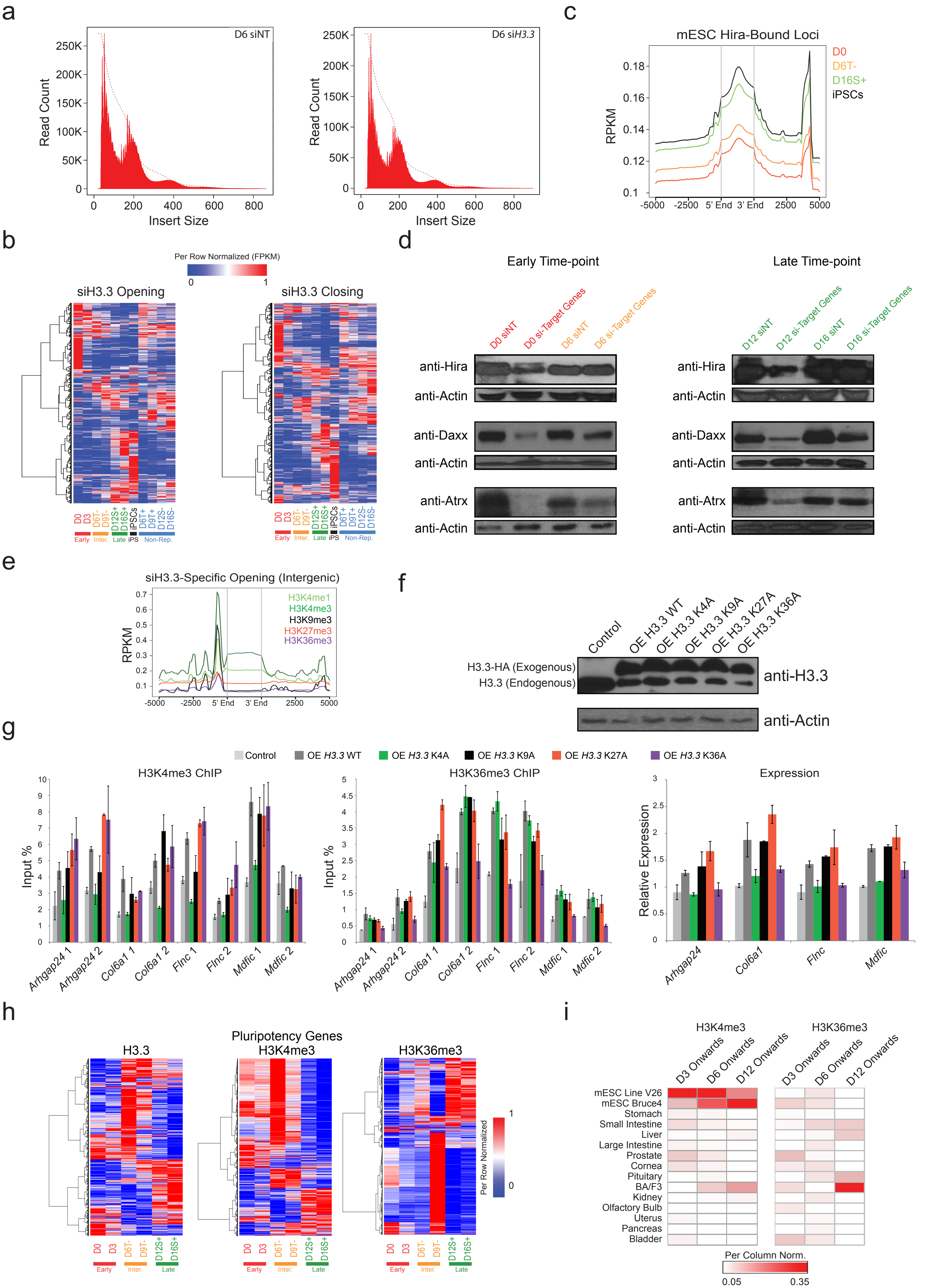

Supplementary Figure 6: (Related to Figure 6). The modification of K4 and K36 residues is required for H3.3 to exhibit its role in cell fate transition.

(a) Tag insert size histogram reveals a nucleosomal pattern for D6 siNT (left) and D6 si*H3.3* (right) ATAC-Seq libraries.

(b) Heatmap revealing the dynamic expression of the genes that exhibit accessibility changes upon H3.3 knockdown (Opening – Left, Closing – Right). The values are per-row normalized FPKM values and colour ranges from dark blue (low expression) to dark red (high expression).

(c) Average enrichment profile of H3.3 reads around the mESC Hira-bound sites. The Y-axis represents average normalized number of fragments mapping to the corresponding regions indicated in the x-axis.

(d) Western blots of the indicated chaperones in day 0 and day 6 (left) day 12 and day 16 reprogramming cells (right). The knock-down of the chaperones took place 2 days prior to reprogramming for day 0 and day 6 blots (early knock-down). For the late knock-down, it was performed at day 9 of cellular reprogramming (right). Non-targeting siRNA constructs (siNT) were used as controls.

(e) Average enrichment profile of mESCs H3K4me1, H3K4me3, H3K36me3, H3K27me3 and H3K9me3 reads around the intergenic regions that show specific opening in D6 siH3.3 ATAC-Seq library. The Y-axis represents average normalized number of fragments mapping to the corresponding regions indicated in the x-axis.

(f) Western blots of overexpressed H3.3 (WT and Mutants) in MEF cells. Empty vectors (EV) were used as control.

(g) Bar charts demonstrating the level of H3K4me3 enrichment (left) and H3K36me3 (middle) on the genes indicated in the x-axis upon the overexpression of H3.3 WT (OE H3.3 WT) and the indicated mutants. Empty expression vectors were used as controls. Y-axis represents the enrichment level in the ChIP sample over input. Bar chart demonstrating the expression levels (right) of the same genes upon the overexpression of WT H3.3 (OE H3.3 WT) and the indicated mutants. Y-axis represents the relative expression levels. Values are mean ± s.e.m from independent replicate experiments (n = 3). Error bars represent standard deviation.

(h) Heatmaps revealing the dynamic enrichment of H3.3 (left), H3K4me3 (middle) and H3K36me3 (right) over pluripotency genes. The values are per-row normalized mapped fragment counts and the colour ranges from dark blue (low enrichment) to dark red (high enrichment).

(i) Heatmaps revealing cell types enrichment from D3, D6 or D12 onwards in H3K4me3 ChIP-Seq (left) and H3K36me3 ChIP-Seq (right). The values are per-column normalized hypergeometric enrichment scores for each cell type. The scale ranges from white (no enrichment) to dark red (highly enriched).

Supplementary Figure 7

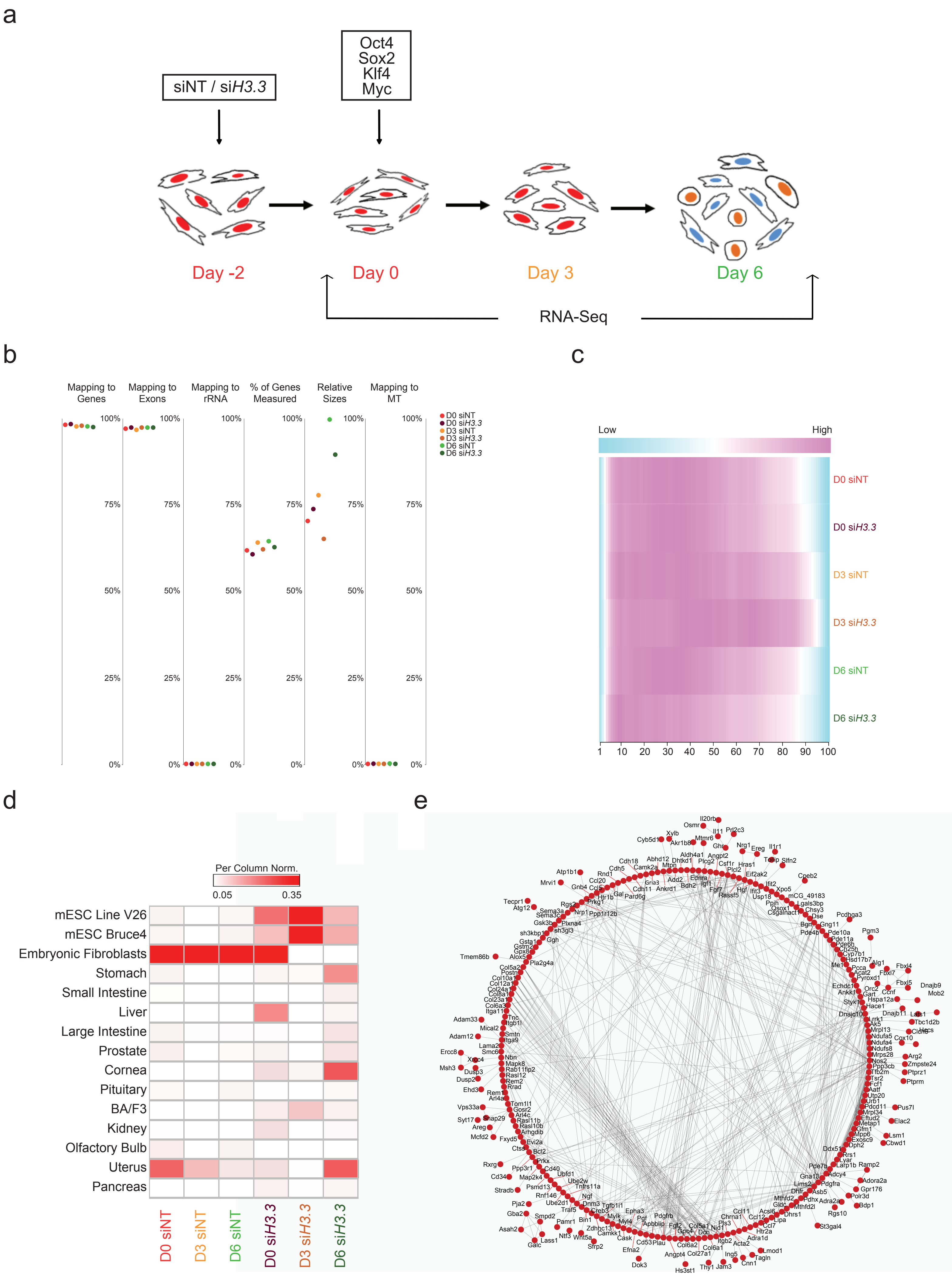

Supplementary Figure 7: (Related to Figure 7). Functional downstream targets of H3.3 safeguard fibroblast lineage.

(a) Schematics of the RNA-Seq libraries prepared in reprogramming cells in which H3.3 had been depleted.

(b) Dot plots demonstrating the mapping to genes %, mapping to exons %, rRNA contamination level, % of genes detected, relative size of libraries to the largest library and the level of MT DNA contamination in the indicated RNA-Seq libraries.

(c) Heatmap revealing the coverage of the indicated RNA-Seq libraries over the genebodies of housekeeping genes. The scale ranges from sky-blue (no coverage) to deep purple (high coverage). The x-axis represents the normalized genebody size percentile.

(d) Heatmap revealing cell types enrichment by genes differentially expressed in the indicated RNA-Seq libraries. The values are per-column normalized hypergeometric enrichment scores for each cell type. The scale ranges from white (no enrichment) to dark red (highly enriched).

(e) Protein-protein interaction network of all functional H3.3 targets.
