# Supplementary material for: Global H3.3 dynamic deposition defines its bimodal role in cell fate transition
Source: Nat Commun. 2018 Apr 18;9:1537. doi: 10.1038/s41467-018-03904-7 (PMC5906632; doi:10.1038/s41467-018-03904-7)
Supplement: Supplementary file 3 — Description of Additional Supplementary Files [file 41467_2018_3904_MOESM3_ESM.pdf]

Description of Additional Supplementary Files

File Name: Supplementary Data 1
Description: Summary of NGS libraries prepared in this study.

File Name: Supplementary Data 2
Description: Dynamic expression values (fpkm) of genes differentially expressed between D0 and iPSCs.

File Name: Supplementary Data 3
Description: Dynamic H3.3 enrichment over genebodies during cellular reprogramming. Values given represent the normalized number of fragments mapping to the genes.

File Name: Supplementary Data 4
Description: Dynamic H3.3 over genebodies during Transdifferentiation. Values given represent the normalized number of fragments mapping to the genes.

File Name: Supplementary Data 5
Description: siH3.3 ATAC-Seq Analysis. Loci Specifically accessible in D6 siH3.3 cells (siH3.3 opening), D6 siNT cells (siH3.3 closing) and common between them (Reprogramming opening)

File Name: Supplementary Data 6
Description: Dynamic H3K4me3 and H3K36me3 enrichment over genebodies during cellular reprogramming. Values given represent the normalized number of fragments mapping to the genes.

File Name: Supplementary Data 7
Description: List of Primers and siRNA constructs used in this study.
